# Time Gain Needed for In-Ambulance Telemedicine: Cost-Utility Model

**DOI:** 10.2196/mhealth.8288

**Published:** 2017-11-24

**Authors:** Alexis Valenzuela Espinoza, Stefanie Devos, Robbert-Jan van Hooff, Maaike Fobelets, Alain Dupont, Maarten Moens, Ives Hubloue, Door Lauwaert, Pieter Cornu, Raf Brouns, Koen Putman

**Affiliations:** ^1^ Interuniversity Center for Health Economics Research Vrije Universiteit Brussel Brussels Belgium; ^2^ Faculty of Medicine and Pharmacy Vrije Universiteit Brussel Brussels Belgium; ^3^ Neurovascular Center Department of Neurology Zealand University Hospital Roskilde Denmark; ^4^ Research Group Clinical Pharmacology and Clinical Pharmacy Vrije Universiteit Brussel Brussels Belgium; ^5^ Department of Neurosurgery Universitair Ziekenhuis Brussel Vrije Universiteit Brussel Brussels Belgium; ^6^ Center for Neurosciences Vrije Universiteit Brussel Brussels Belgium; ^7^ Department of Emergency Medicine Universitair Ziekenhuis Brussel Vrije Universiteit Brussel Brussels Belgium; ^8^ Research Group on Emergency and Disaster Medicine Vrije Universiteit Brussel Brussels Belgium; ^9^ Department of Neurology ZorgSaam Hospital Terneuzen Netherlands

**Keywords:** telemedicine, prehospital, stroke, cost effectiveness

## Abstract

**Background:**

Stroke is a very time-sensitive pathology, and many new solutions target the optimization of prehospital stroke care to improve the stroke management process. In-ambulance telemedicine, defined by live bidirectional audio-video between a patient and a neurologist in a moving ambulance and the automated transfer of vital parameters, is a promising new approach to speed up and improve the quality of acute stroke care. Currently, no evidence exists on the cost effectiveness of in-ambulance telemedicine.

**Objective:**

We aim to develop a first cost effectiveness model for in-ambulance telemedicine and use this model to estimate the time savings needed before in-ambulance telemedicine becomes cost effective.

**Methods:**

Current standard stroke care is compared with current standard stroke care supplemented with in-ambulance telemedicine using a cost-utility model measuring costs and quality-adjusted life-years (QALYs) from a health care perspective. We combine a decision tree with a Markov model. Data from the UZ Brussel Stroke Registry (2282 stroke patients) and linked hospital claims data at individual level are combined with literature data to populate the model. A 2-way sensitivity analysis varying both implementation costs and time gain is performed to map the different cost-effective combinations and identify the time gain needed for cost effectiveness and dominance. For several modeled time gains, the cost-effectiveness acceptability curve is calculated and mapped in 1 figure.

**Results:**

Under the base-case scenario (implementation cost of US $159,425) and taking a lifetime horizon into account, in-ambulance telemedicine is a cost-effective strategy compared to standard stroke care alone starting from a time gain of 6 minutes. After 12 minutes, in-ambulance telemedicine becomes dominant, and this results in a mean decrease of costs by US –$30 (95% CI –$32 to –$29) per patient with 0.00456 (95% CI 0.00448 to 0.00463) QALYs on average gained per patient. In over 82% of all probabilistic simulations, in-ambulance telemedicine remains under the cost-effectiveness threshold of US $47,747.

**Conclusions:**

Our model suggests that in-ambulance telemedicine can be cost effective starting from a time gain of 6 minutes and becomes a dominant strategy after approximately 15 minutes. This indicates that in-ambulance telemedicine has the potential to become a cost-effective intervention assuming time gains in clinical implementations are realized in the future.

## Introduction

Stroke is a very time-sensitive pathology, and many new solutions target the optimization of prehospital stroke care to improve the stroke management process [1]. One approach to speed up the stroke care process is the deployment of mobile stroke units (MSUs) that focus on the prehospital diagnosis and intravenous administration of recombinant tissue plasminogen activator (IVT) [2]. This is achieved by bringing the computed tomography (CT) scan to the patient, and time gains of 15 minutes between emergency call to IVT have been realized using this method [3]. In-ambulance telemedicine is another promising approach to reduce delays of the in-hospital stroke response by gathering and transferring relevant diagnostic information while the patient is underway to the hospital and therefore facilitating the clinical decision making on performing a CT scan and treatment initiation [4]. Recent progress in mobile connectivity enables virtually every ambulance to be equipped with telemedicine solutions, and several projects confirm the medical interest in this approach [4-8]. In-ambulance telemedicine allows head-to-toe examination of each patient through bidirectional audio-video communication between the ambulance and a remote teleconsultant and the secure transfer of medical data during emergency transportation of patients to a care facility. Pilot studies on stroke patients have shown that 24/7 in-ambulance telemedicine support is feasible, and stroke-specific information can be collected and communicated to the in-hospital team during emergency ambulance transportation [4,5]. The use of in-ambulance telemedicine is well accepted by patients and emergency personnel [5,9]. The combination of prehospital triage, early notification of the receiving in-hospital team, and communication of stroke-specific information by a remote stroke expert while the patient is being transported to the hospital has the potential to speed up the stroke diagnosis and treatment [4]. A time gain of 20 minutes has the potential to improve the probability of a favorable outcome after intravenous thrombolysis by 2.3% in a mixed stroke population [10] and is associated with reduced in-hospital mortality, lower risk of symptomatic intracranial hemorrhage, increased chance of independency at discharge, and increased probability to be discharged home [11]. Novel endovascular therapies are also highly time sensitive, and probabilities of favorable outcome increase relevantly when delays to treatment initiation decrease [12].

Currently, no evidence exists on the cost effectiveness of in-ambulance telemedicine. We aim to develop a model which predicts the potential costs and benefits associated with this new in-ambulance telemedicine approach. Consequently, this model allows identification of the minimum time gain that is needed before in-ambulance telemedicine becomes cost effective.

## Methods

### Model Description

**Figure 1:**
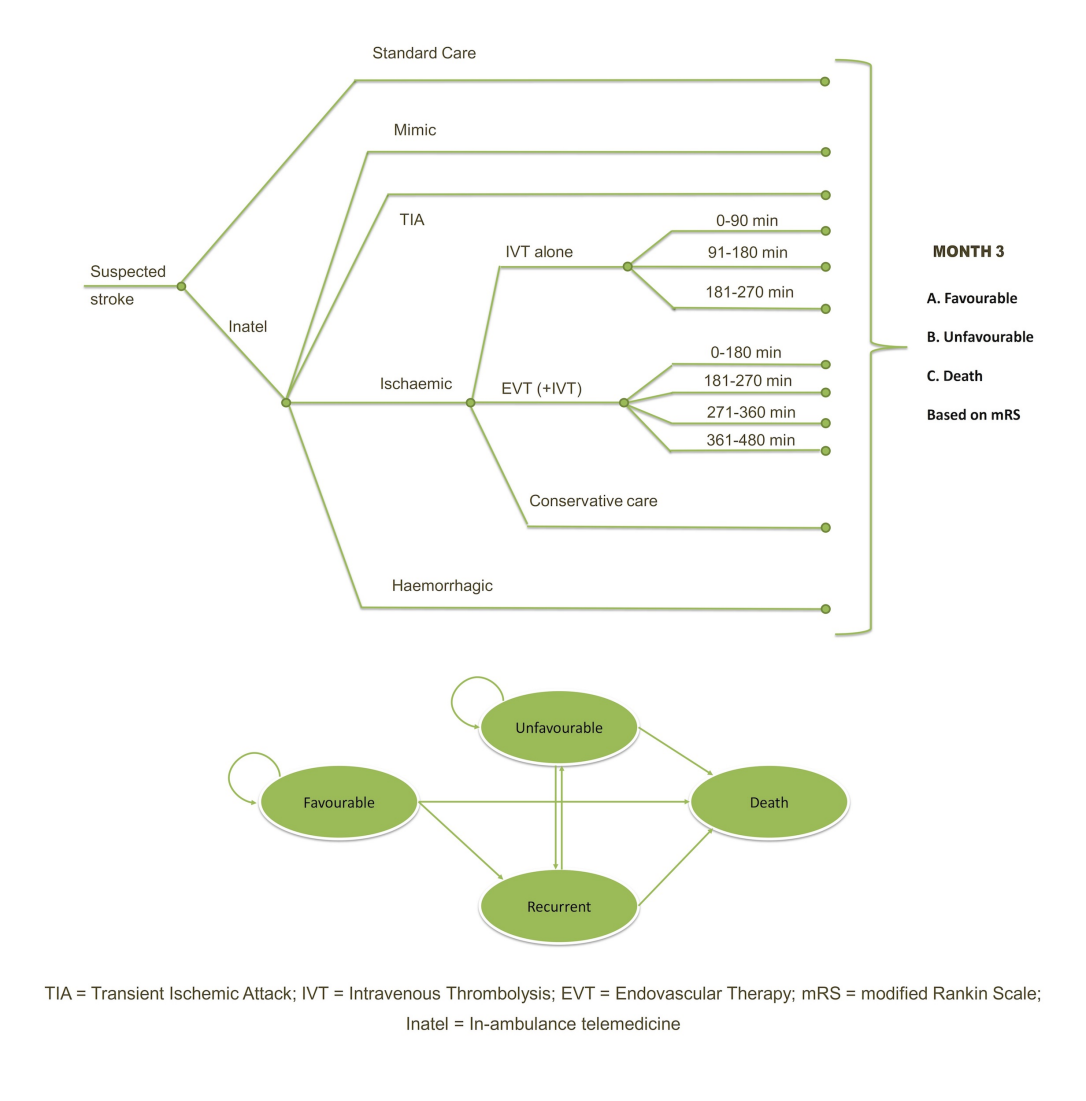
Decision tree and Markov model for in-ambulance telemedicine for suspected stroke patients.

Current standard stroke care is compared with current standard stroke care supplemented with in-ambulance telemedicine using a cost-utility model measuring costs and quality adjusted life-years (QALYs) from a health care perspective. We combine a decision tree model (3 months) with a Markov model using a lifetime horizon (Figure 1). One possible implementation of the intervention (in-ambulance telemedicine) was previously described [4]. In-ambulance telemedicine allows the automated transmission of vital parameters (heart rate, blood oxygen saturation, and systolic and diastolic blood pressure), glycemia, electronic patient identification, functional assessments, and prehospital notification of the in-hospital team. Teleconsultants are not required to remain in the hospital to ensure 24/7 coverage because telemedicine support can be provided from any location with access to the Internet. A report of the teleconsultation containing all available information is immediately sent to the in-hospital team. Teleconsultants and ambulance personnel are trained to adequately use the telemedicine system. The in-hospital team is taught how to securely access and interpret the teleconsultation report.

All patients with suspicion of acute stroke are included in the model and are divided into 4 main categories: (1) stroke mimic, (2) transient ischemic attack (TIA), (3) ischemic stroke, and (4) hemorrhagic stroke. Ischemic strokes are divided into 3 treatment groups: (1) intravenous administration of IVT alone, (2) intravenous administration of IVT in combination with endovascular treatment (EVT), or (3) conservative care. Patients in the in-ambulance telemedicine model can either receive in-ambulance telemedicine on top of standard stroke care or standard stroke care alone. This additional arm accounts for the missed opportunities related to the accuracy of dispatchers to recognize a stroke and the proportion of patients not transported by an ambulance equipped with telemedicine technology.

To effectively model the time-sensitive nature of IVT with and without EVT, the treatment effect per time interval is modeled. For IVT alone, we assume that patients are treated up to 4.5 hours after stroke onset [13]. In combination with EVT, a positive effect is observed until 8 hours after stroke onset [12].

The comparator of standard stroke care is based on the performance indicators of Universitair Ziekenhuis Brussel (UZ Brussel), the university hospital of the Free University of Brussels (VUB), between 2011 and 2014. In this center, in-hospital prealerting of potential stroke patients and streamlined in-hospital workflows are part of standard medical practice [4].

### Probabilities and Utilities

For each end node in the decision tree, the modified Rankin Scale (mRS) at month 3 is used to classify patients into 3 categories: (1) favorable outcome (mRS 0-2), (2) unfavorable outcome (mRS 3-5), and (3) death (mRS 6). Data from the UZ Brussel Stroke Registry (2282 suspected stroke patients admitted to the UZ Brussel Stroke Unit between February 2009 and February 2014) are combined with literature data to populate the model for standard care (Table 1 and Multimedia Appendix 1). Each mRS group is associated with utilities. The cycle length of the Markov model is 1 year with a lifetime horizon. Patients enter the Markov model in the favorable or unfavorable state and transition to their current state, to a death state, or to a recurrent stroke state. Transition after a recurrent stroke is limited to the unfavorable or death state.

**Table 1:** Parameters used to populate the standard care model.

Parameter					Base-case value (probability)	Source
**Probabilities**						
	**After Suspected Stroke**					
		Stroke mimic			0.22	Stroke Registry UZ Brussel^a^
		TIA^b^			0.05	Sheppard et al [14]
		Ischemic stroke			0.66	Stroke Registry UZ Brussel
		Hemorrhagic stroke			0.07	Stroke Registry UZ Brussel
	**After ischemic stroke**					
		IVT^c^			0.15	Stroke Registry UZ Brussel
		EVT^d^			0.05	Vanacker et al [15]
		Conservative treatment			0.8	Stroke Registry UZ Brussel
	**After ischemic stroke and IVT**					
		0-90 min			0.12	OTT^e^ distributions from Lees et al [16]
		91-180 min			0.24	OTT distributions from Lees et al [16]
		181-270 min			0.64	OTT distributions from Lees et al [16]
	**After ischemic stroke and EVT**					
		0-180 min			0.16	Campbell et al [17]
		181-270 min			0.21	Campbell et al [17]
		271-360 min			0.41	Campbell et al [17]
		361-480 min			0.23	Campbell et al [17]
	**After stroke mimic**					
		Favorable			0.78	Stroke Registry UZ Brussel
		Unfavorable			0.04	Stroke Registry UZ Brussel
		Death			0.17	Stroke Registry UZ Brussel
	**After TIA**					
		Favorable			0.82	Stroke Registry UZ Brussel
		Unfavorable			0.14	Stroke Registry UZ Brussel
		Death			0.04	Stroke Registry UZ Brussel
	**After ischemic stroke and conservative treatment**					
		Favorable			0.48	Wardlaw et al [18]
		Unfavorable			0.04	Wardlaw et al [18]
		Death			0.12	Wardlaw et al [18]
	**After ischemic stroke and IVT**					
		**0-90 min**				
			Favorable		0.7	Lees et al [16]
			Unfavorable		0.18	Lees et al [16]
			Death		0.12	Lees et al [16]
		**91-180 min**				
			Favorable		0.59	Lees et al [16]
			Unfavorable		0.29	Lees et al [16]
			Death		0.12	Lees et al [16]
						
		**181-270 min**				
			Favorable		0.55	Lees et al [16]
			Unfavorable		0.33	Lees et al [16]
			Death		0.12	Lees et al [16]
	**After ischemic stroke and EVT**					
		**0-180 min**				
			Favorable		0.78	Multimedia Appendix 1, Fransen et al [12]
			Unfavorable		0.1	Multimedia Appendix 1, Fransen et al [12]
			Death		0.12	Multimedia Appendix 1, Fransen et al [12]
		**181-270 min**				
			Favorable		0.70	Multimedia Appendix 1, Fransen et al [12]
			Unfavorable		0.18	Multimedia Appendix 1, Fransen et al [12]
			Death		0.12	Multimedia Appendix 1, Fransen et al [12]
		**271-360 min**				
			Favorable		0.59	Multimedia Appendix 1, Fransen et al [12]
			Unfavorable		0.29	Multimedia Appendix 1, Fransen et al [12]
			Death		0.12	Multimedia Appendix 1, Fransen et al [12]
		**361-480 min**				
			Favorable		0.51	Multimedia Appendix 1, Fransen et al [12]
			Unfavorable		0.29	Multimedia Appendix 1, Fransen et al [12]
			Death		0.12	Multimedia Appendix 1, Fransen et al [12]
	**After hemorrhagic stroke**					
		Favorable			0.44	Anderson et al [19]
		Unfavorable			0.44	Anderson et al [19]
		Death			0.12	Anderson et al [19]
**Utilities**						
	Utility in the favorable state (mRS^f^ 0-2)				0.74	Dorman et al [20]
	Utility in the unfavorable state (mRS 3-5)				0.38	Dorman et al [20]
	Utility in the death state				0	
	Utility in the recurrent state				0.34	Morris et al [21]
**Markov transitions**						
	Probability recurrent stroke				0.05	Sandercock et al [22]
	Increased mortality risk after recurrent stroke				0.25	Sandercock et al [22]
	Multiplier for age-specific mortality among stroke patients				2.5	Sandercock et al [22]
	**Mortality after stroke**					
		70-74 years			0.05	Belgian mortality statistics corrected for age-specific mortality among stroke patients
		75-79 years			0.08	Belgian mortality statistics corrected for age-specific mortality among stroke patients
		80-84 years			0.14	Belgian mortality statistics corrected for age-specific mortality among stroke patients
		85-89 years			0.26	Belgian mortality statistics corrected for age-specific mortality among stroke patients
		90+ years			0.45	Belgian mortality statistics corrected for age-specific mortality among stroke patients
**Other**						
	Average age of stroke patients				73	Thijs et al [23]
	Discount rate for costs				0.03	KCE^g^ [24]
	Discount rate for utilities				0.015	KCE [24]

^a^UZ Brussel: Universitair Ziekenhuis Brussel.

^b^TIA: transient ischemic attack.

^c^IVT: intravenous administration of recombinant tissue plasminogen activator.

^d^EVT: endovascular treatment.

^e^OTT: onset to treatment time.

^f^mRS: modifed Rankin Scale.

^g^KCE: Belgian Health Care Knowledge Centre.

### Costs

For the costs per treatment arm, we link hospital data and emergency claims data (including all payer costs) at the individual patient level (Multimedia Appendices 2 and 3). Claims for drugs, clinical biology, medical imaging, physicians’ honoraria, other claims charged to patients (copayments), and health insurances were included. To calculate the total hospital cost, a fixed day price was added according to the year of admission. This fixed day price covers the financing of nonmedical hospital activities. For this study, the weighted average per diem prices (across Belgian hospitals) was used [24]. All costs are expressed in and discounted to 2014, and Euro and US $ equivalents are calculated using the average 2014 exchange rate (€1=US $1.329). We do not model any productivity loss, as the average age of our patient cohort is 73 years. The cost-effectiveness threshold is set at US $47,747 (€35,927), the gross domestic product per capita of Belgium in 2014.

### Impact of In-Ambulance Telemedicine

The impact of in-ambulance telemedicine on top of standard care is modeled by assuming an average time gain ranging from 5 to 60 minutes (Table 2 and Multimedia Appendix 4). This influences the treatment of stroke patients in 2 fundamental ways. First, probabilities of a positive outcome after treatment with IVT and/or EVT increase, since more patients are shifted into an earlier time window. Second, more patients can be treated with IVT and/or EVT as more patients shift into the applicable time windows, 4.5 hours and 8 hours, respectively, after symptom onset. Costs of the intervention are modeled by adding a fee per teleconsultation and a fixed fee per ambulance in which a telemedicine device is installed (Table 2 and Multimedia Appendix 5). Training of all stakeholders and mobile connectivity costs are included in the telemedicine installation cost.

Based on previous in-ambulance telemedicine pilot studies [4,5], we assume that 150 patients can be treated with 1 ambulance on a yearly basis, resulting in 3 ambulances to be equipped with the telemedicine technology for a patient cohort of 1000 suspected stroke patients (390 patients receiving in-ambulance telemedicine). All other parameters are assumed equal to standard medical stroke care.

### Model Output

Costs and QALYs are used to calculate the incremental cost-effectiveness ratio (ICER) after 3 months (decision tree only) and after a lifetime horizon (decision tree plus Markov model). Time gain after in-ambulance telemedicine is varied between 0 and 60 minutes, and cost for the implementation of in-ambulance telemedicine is varied between 50% and 400% of baseline cost in a 2-way sensitivity analysis, mapping the ICER for all combinations of both variables. Based on this analysis, we select the time gain for which in-ambulance telemedicine becomes cost effective. For this time gain, we perform a 1-way sensitivity analysis, varying all input parameters between 70% and 130% of their deterministic value and ranking the parameters according to the highest interval between calculated outcome parameters (both cost and QALYs are calculated). A probabilistic sensitivity analysis is applied using Monte Carlo simulations with 5000 bootstraps to account for the uncertainty around the input parameters and assess the robustness of the model. The cost-effectiveness acceptability curve is constructed for 5, 10, 15, and 30 minutes of time gain (1000 bootstraps). The health economic model was built and runs in Excel (Microsoft Office Professional Plus 2013, Microsoft Corp), and the UZ Brussel Stroke Registry was analyzed using Stata MP 13.0 for Windows (StataCorp LLC). The study was approved by the UZ Brussel ethical committee, and the model was validated by MF.

**Table 2:** Adapted parameters for in-ambulance telemedicine under 12 minutes time gain on average per patient and additional costs.

Parameter				Base-case value		Source/assumption
**Probabilities**						
	**After suspected stroke**					
		Standard care		0.61		Multimedia Appendix 4
		In-ambulance telemedicine		0.39		Multimedia Appendix 4
	**After ischemic stroke**					
		IVT^a^		0.19		Multimedia Appendix 4
		EVT^b^		0.07		Multimedia Appendix 4
		Conservative treatment		0.73		Multimedia Appendix 4
	**After ischemic stroke and IVT**					
		0-90 min		0.15		Multimedia Appendix 4
		91-180 min		0.29		Multimedia Appendix 4
		181-270 min		0.56		Multimedia Appendix 4
	**After ischemic stroke and EVT**					
		0-180 min		0.19		Multimedia Appendix 4
		181-270 min		0.23		Multimedia Appendix 4
		271-360 min		0.38		Multimedia Appendix 4
		361-480 min		0.19		Multimedia Appendix 4
**Costs, US $ (€)**						
	Cost per teleconsultation			142.89 (107.52)		Multimedia Appendix 5
	Cost of installation of 1 telemedicine device			29,011 (26,000)		Offer from Zebra Academy
	Estimated total cost for in-ambulance telemedicine for 390 treated patients in 1 year			159,425 (119,959)		Multimedia Appendix 5
Number of patients that can be treated with 1 device in 1 year				150		Activation rates of the PreSSUB-I^c^ trial [4]

^a^IVT: intravenous administration of recombinant tissue plasminogen activator.

^b^EVT: endovascular treatment.

^c^PreSSUB-I: Prehospital Study at the Universitair Ziekenhuis Brussel I.

## Results

### Base-Case

**Figure 2:**
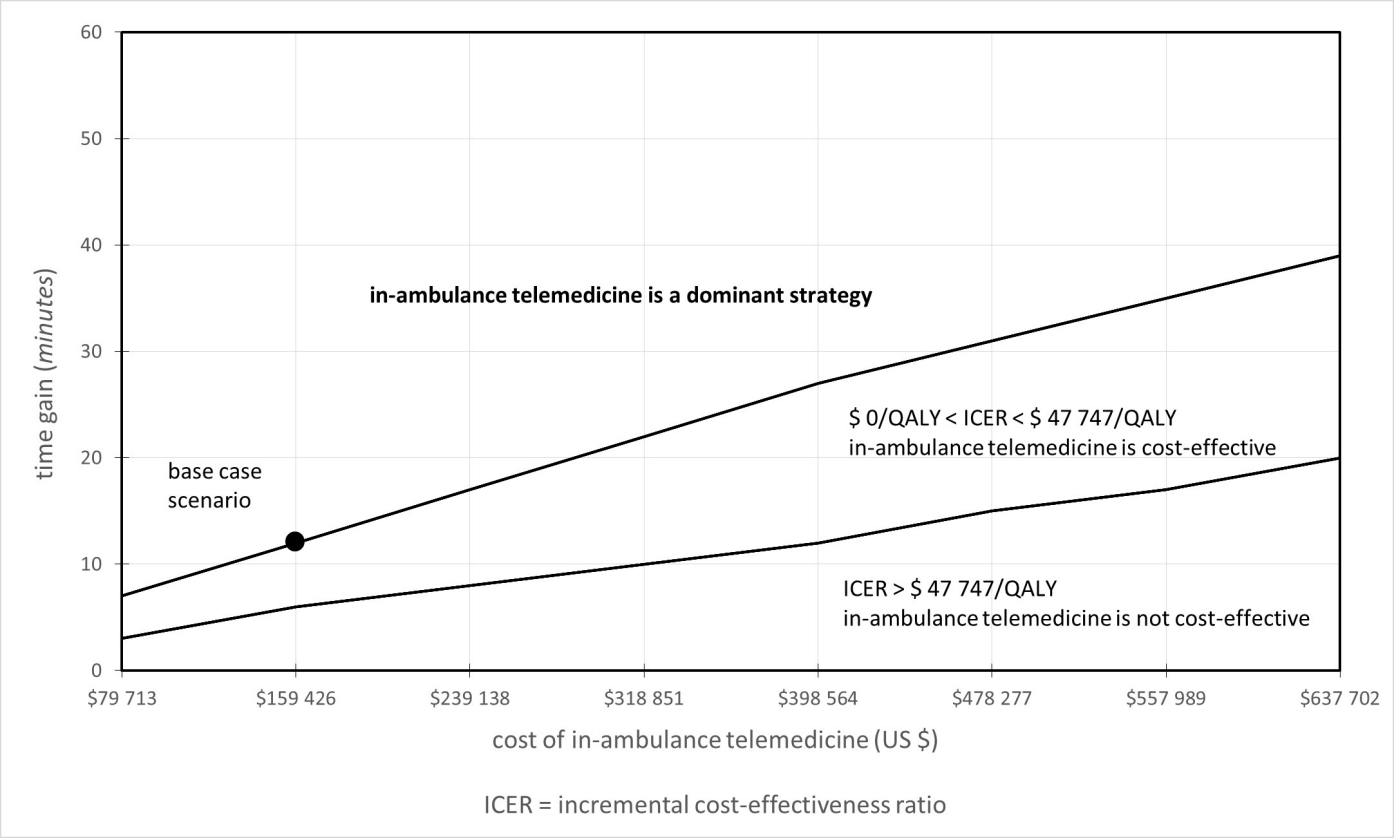
Two-way sensitivity analysis for in-ambulance telemedicine compared to standard care. Implementation costs are varied between 0.5 and 4 times the base case cost and time-gain is varied between 0 and 60 minutes.

Under the base-case scenario (implementation cost of US $159,425) and taking a lifetime horizon into account, in-ambulance telemedicine is a cost-effective strategy compared to standard stroke care alone, starting from a time gain of 6 minutes (Figure 2).

After 12 minutes, in-ambulance telemedicine becomes a dominant strategy over standard best medical practice (Table 3). In a cohort of 1000 patients, 4.9 QALYs are gained (0.005 QALY/patient) and US $4040 (€3040) in long-term costs are avoided (–$4/patient). The savings of earlier stroke treatment outweigh the cost for implementation of in-ambulance telemedicine (cost equals US $159,425 [€119,959] for 390 patients receiving in-ambulance telemedicine) and higher utilization rates of specific stroke treatments (IVT and EVT). After 3 months, unfavorable outcome is avoided in 2.42 additional patients, resulting in long-term savings for society.

Not taking into account these long-term savings, in-ambulance telemedicine yields an ICER of US $201,557/QALY (€151,660/QALY) after 3 months. This incremental cost per saved QALY is explained by the cumulative cost of the intervention and the costs associated with more IVT and EVT after implementation of in-ambulance telemedicine.

### One-Way Sensitivity Analysis

One-way sensitivity analysis at 12 minutes time gain reveals that parameters involving outcome of ischemic stroke have the largest impact on calculated costs and QALYs (Multimedia Appendices 6-9). For likelihood of unfavorable outcome after conservative care, the incremental cost/patient varies between US –$252 (–€190) (130%) and US $244 (€184) (70%); for favorable outcome after conservative care, the incremental QALY/patient varies between 0.013 (70%) and –0.003 (130%). This is not surprising given the model’s rationale (shift from conservative care to IVT) and the time-sensitive nature of IVT. We note, however, that these parameters are taken from the analysis of pooled randomized controlled trials (RCT) [16,18], and the time-sensitive nature of IVT has been confirmed in larger clinical populations [11,25]. The proportion of patients receiving standard care (vs in-ambulance telemedicine) influences the outcome parameters of the model, indicating that regions with more ambulances equipped with telemedicine, with a higher ability of ambulance dispatchers to recognize a stroke, and with a higher proportion of patients being transported by ambulance will benefit more from in-ambulance telemedicine.

### Two-Way Sensitivity Analysis

Under 4 times baseline costs (>US $500,000 implementation costs), in-ambulance telemedicine becomes cost effective after 19 minutes and is dominant after 39 minutes (Figure 2). For lower cost implementations (<US $70,000), in-ambulance telemedicine can be cost effective after 3 minutes and dominant after 7 minutes of achieved time gain.

### Probabilistic Results

**Figure 3:**
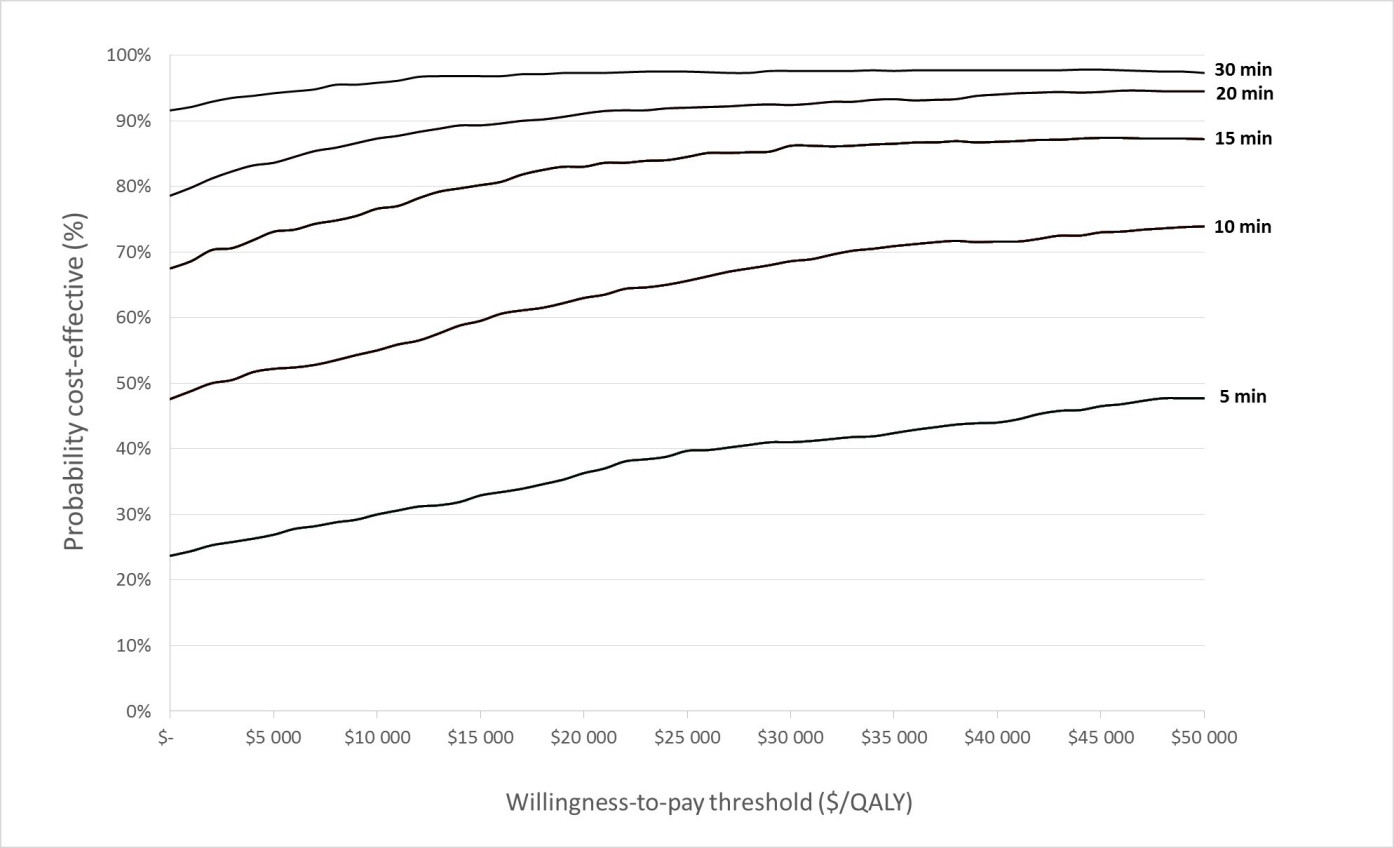
The cost-effectiveness acceptability curve for in-ambulance telemedicine compared to standard care is calculated for 1000 bootstraps per time interval (5, 10, 15, 20, and 30 minutes).

The cost-effectiveness acceptability curves (Figure 3) reveal that under probabilistic analysis more than 90% of simulations are cost effective at the threshold of US $47,747, starting from 15 minutes time gain. This number drops below 80% under a scenario of 10 minutes time gain.

**Table 3:** Deterministic costs and quality-adjusted life-years after 3 months and after a lifetime horizon under 12 minutes time gain.

Cohort of 1000 patients	3 months		Lifetime horizon	
	Total costs ($)	Outcome (QALY^a^)	Total costs ($)	Outcome (QALY)
Standard care	21,530,867	537.6	92,068,697	3649.8
In-ambulance telemedicine	21,706,449	538.4	92,064,657	3654.8
Difference	175,582	0.9	–4040	4.9
ICER^b^, $/QALY	201,557		–817	

^a^QALY: quality-adjusted life-year.

^b^ICER: incremental cost-effectiveness ratio.

## Discussion

### Principal Findings

We report on the first ever comprehensive in-ambulance telemedicine cost-utility model combining a decision tree with a Markov model, using detailed cost information per treatment arm. In-ambulance telemedicine is dominant from a health care payer perspective starting from 12 minutes time gain.

Two previous publications describe health economic aspects of MSUs for improvement of prehospital stroke care [26,27]. Both models use the same source for calculation of improved outcome following faster IVT [16]. We applied the same methodology in our model by calculating the absolute risk difference based on the reported numbers needed to treat.

### Limitations

No impact of in-ambulance telemedicine on mortality was modeled, even though evidence exists that earlier stroke treatment reduces in-hospital mortality [11]. We chose not to model mortality because sufficiently specific information on mortality is not available in RCTs and because it may be unlikely that our population of 2200 patients would be sufficiently powered to model a possible impact on mortality.

The impact of in-ambulance telemedicine can vary greatly from 1 hospital or region to another depending on the standard quality and speed of care. Streamlining in-hospital workflows will always be a crucial part of a successful in-ambulance telemedicine implementation and can influence costs and potential benefit. For example, realized time gains could be more modest if prehospital notification through mobile phones or tablets is already part of current practice.

The major limitation of the presented model lies in the absence of information from RCTs evaluating the effects of in-ambulance telemedicine on costs and patient outcome. This drawback was addressed by implementing only solid criteria originating from RCTs for the outcome parameters. However, combining data from multiple trials is not without risk, and the results of this model should be interpreted carefully.

### Other Considerations

An advantage of in-ambulance telemedicine is the limited amount of additional resources needed from the hospital. If state-of-the art stroke care is available, no additional staff may be needed and existing ambulances can readily be equipped with the technology. However, depending on the catchment area and the number of stroke patients supported via in-ambulance telemedicine, additional teleconsultants on call may be needed. Although this could increase the organizational cost of in-ambulance telemedicine, more patients would be treated with in-ambulance telemedicine, which would further decrease the cost per patient and improve outcome. Training is required but was included in the cost of the telemedicine implementation.

Total implementation costs will decrease when in-ambulance telemedicine technology becomes more widely available. Lower cost alternatives such as tablet-based approaches are currently being investigated for remote stroke severity assessment in driving ambulances [6,7].

Widespread implementation of in-ambulance telemedicine will not only depend on its (cost-)effectiveness but also on the creation of the required legal framework. In Belgium, currently consultations are only officially recognized if face-to-face contact between the patient and treating physician occurs. Other issues include clear regulations for reimbursement and liability.

Our analysis only takes benefits of the expected time gain into account. Other benefits of in-ambulance telemedicine include a lower risk of stroke misdiagnosis and consequently missed opportunities for treatment with IVT or EVT and triage of patients to inadequate facilities [28]. Estimates of missed stroke diagnosis by emergency personnel range from 22% to 47%, indicating the potential for in-ambulance telemedicine to curtail the risk of misdiagnosis [29,30]. Reducing missed opportunities was not modeled here to avoid double counting when combined with faster treatment effects.

We excluded the implementation of rapid blood pressure lowering for hemorrhagic stroke patients in our model, even though indications exist on the benefit of this approach [19]. In-ambulance telemedicine could increase the proportion of patients receiving rapid blood pressure lowering, resulting in an underestimation of the potential cost effectiveness.

Further, expert prehospital care may help avoid secondary brain damage, as the teleconsultant can support the ambulance personnel in obtaining and maintaining homeostasis during ambulance transportation through optimal application of the standard operating procedures for airway protection, blood oxygen saturation, arterial blood pressure, heart rate, cardiac arrhythmia, decreased level of consciousness, dysglycemia, and other supportive measures (eg, antiemetics, analgesics).

The combination of in-ambulance telemedicine with MSUs is another interesting approach that could further decrease costs of the MSU as it would avoid sending highly trained physicians into the field for each individual patient. This approach is feasible, and preliminary analysis has shown that median time savings of 23 minutes between alarm-to-CT times can be attained when compared to standard care [31].

Recent clinical trials showing impressive benefits from EVT [32-34] herald a new era in acute ischemic stroke care. This highly effective treatment is resulting in a paradigm shift toward optimization of prehospital stroke diagnosis, identification of suitable candidates for IVT and EVT, and patient triage to appropriate centers [35]. A care model that avoids secondary transportations of stroke patients from primary stroke centers to comprehensive stroke centers for EVT is expected to result in better patient outcome for at least 0.2% of the patient cohort [10]. We did not take these effects of in-ambulance telestroke into account as their supportive evidence currently is insufficient to allow robust modeling. This probably results in an underestimation of the benefits yielded by in-ambulance telemedicine.

In-ambulance telemedicine has the potential to improve the organization of care for other medical emergencies, further strengthening the cost-effectiveness potential of this technology. The use of tablet computers as a support system for general emergency medical services and better patient triage have shown a decrease in transportation times by ambulances, showing the possibilities for further innovation in emergency care organization and delivery using telemedicine and mobile health solutions [36].

We believe that this positive health economic evaluation can inspire decision makers in hospitals and governments to actively pursue the implementation of and further research on in-ambulance telemedicine.

### Conclusions

In-ambulance telestroke is highly cost effective from a health care perspective, resulting in more QALYs and less costs starting from a realized time gain of 12 minutes. The model is not directly based on results from RCTs on the effects of in-ambulance telemedicine, and trials to further crystalize these effects in various care models are needed. Support from governments and hospitals to facilitate implementation in clinical practice is indispensable and can be justified by the dominant cost effectiveness of in-ambulance telemedicine under several scenarios both in terms of implementation costs and time gain.

## Appendix

Multimedia Appendix 1 Details on all parameters used to populate the standard-care model.

Multimedia Appendix 2 Details on the costs used in the model.

Multimedia Appendix 3 Details on 1-year poststroke costs.

Multimedia Appendix 4 Adapted probabilities for in-ambulance telemedicine.

Multimedia Appendix 5 Costs related to in-ambulance telemedicine.

Multimedia Appendix 6 Tornado input probabilities—incremental cost per patient.

Multimedia Appendix 7 Tornado input probabilities—incremental quality-adjusted life-years per patient.

Multimedia Appendix 8 Tornado input costs, utilities, and other parameters—incremental cost per patient.

Multimedia Appendix 9 Tornado input costs, utilities, and other parameters—incremental quality-adjusted life-years per patient.

## Abbreviations

CT: computed tomography
EVT: endovascular treatment
ICER: incremental cost-effectiveness ratio
INNOVIRIS: Brussels Institute for Research and Innovation
IVT: intravenous administration of recombinant tissue plasminogen activator
KCE: Belgian Health Care Knowledge Centre
mRS: modified Rankin Scale
MSU: mobile stroke unit
OTT: onset to treatment time
QALY: quality-adjusted life-year
TIA: transient ischemic attack
UZ Brussel: Universitair Ziekenhuis Brussel
VUB: Free University of Brussels
