# Supplementary material for: Time Gain Needed for In-Ambulance Telemedicine: Cost-Utility Model
Source: JMIR Mhealth Uhealth. 2017 Nov 24;5(11):e175. doi: 10.2196/mhealth.8288 (PMC5722977; doi:10.2196/mhealth.8288)
Supplement: Multimedia Appendix 1 [file mhealth_v5i11e175_app1.pdf]

Multimedia Appendix 1 - Details on all parameters used to populate the standard-care model

Parameter	Base-Case Value	Calculation/ Assumption/ Source	Distribution	Alpha	Beta
Probabilities					
After Suspected Stroke<br>mimic / TIA / Isch / Haem	0,22 / 0,05* / 0,66 / 0,07	UZ Brussel Stroke Registry<br>*Sheppard et al. 2016[1] – TIA's in UZ Brussel Stroke Registry equaled 20% - excess TIA's were transitioned to Isch group.	Dirichlet	217 / 48 / 664 / 72	1 / 1 / 1 / 1
after Isch<br>IVT / EVT / Cons	0,15 / 0,05* / 0,8	*According to Vanacker et al. 2016[2] 10% of Isch stroke patients are eligible for EVT.<br>20% IVT+ EVT (UZ Brussel Stroke Registry).<br>Assume 50% of eligible patients is treated under standard care.	Dirichlet	98 / 35 / 530	1 / 1 / 1 / 1
after Isch & IVT<br>0-90 min / 91-180 min / 181-270 min	0,12 / 0,24 / 0,64	OTT distributions from Lees et al. 2010[3]	Dirichlet	10 / 19 / 50 / 34	1 / 1 / 1 / 1
after Isch & EVT<br>0-180 min / 181-270 min / 271-360 min / 361-480 min	0,16 / 0,21 / 0,41 / 0,23	Campbell et al. 2016[4]<br>Time between start IVT and onset EVT equals 68 min.<br>Equal distribution as after Isch & IVT + shift of 68 min.<br>To apply time shift we assume patients are distributed equally in a time interval. A shift of 30 minutes in a time interval of 90 minutes, means that 1/3th (30min/90min) of patients within that time interval shift.	Dirichlet	3 / 4 / 7 / 4	1 / 1 / 1 / 1
after Mimic<br>Fav / Unfav / Death	0,78 / 0,04 / 0,17	UZ Brussel Stroke Registry	Dirichlet	170 / 9 / 38	1 / 1 / 1 / 1
after TIA<br>Fav / Unfav / Death	0,82 / 0,14 / 0,04	UZ Brussel Stroke Registry	Dirichlet	39 / 7 / 2	1 / 1 / 1 / 1
after Isch & Cons<br>Fav / Unfav / Death	0,48 / 0,40 / 0,12	Wardlaw et al. 2012[5] – control group	Dirichlet	254 / 213 / 64	1 / 1 / 1 / 1
after Isch & IVT &<br>0-90 min<br>91-180 min<br>181-270 min<br>Fav / Unfav / Death	0,7 / 0,18 / 0,12<br>0,59 / 0,29 / 0,12<br>0,55 / 0,33 / 0,12	Lees et al. 2010[3]<br>Calculation of ARR for mRS 0-1 based on NNT (ARR=1/NTT)<br>0,22 / 0,11 / 0,07 / 0,04<br>Favourable outcome (mRS0-2) calculated based on probabilities in after Isch & Cons & Fav (0,48) + ARR. Assume that ARR for mRS0-1 equals ARR for mRS0-2.	Dirichlet<br>Dirichlet<br>Dirichlet	8 / 2 / 1<br>11 / 6 / 2<br>28 / 17 / 6	1 / 1 / 1 / 1<br>1 / 1 / 1 / 1<br>1 / 1 / 1 / 1
after Isch & EVT &	0,78 / 0,1 / 0,12	Fransen et al. 2016[6]<br>Calculation of ARR for mRS 0-2 based on ARD	Dirichlet<br>Dirichlet	2 / 0,3 / 0,4	1 / 1 / 1 / 1

0-181 min	0,70 / 0,18 / 0,12	ARR = ARD	Dirichlet	3 / 0,7 / 0,4	1 / 1 / 1 / 1
181-270 min	0,59 / 0,29 / 0,12	0,30 / 0,22 / 0,11 / 0,03	Dirichlet	4 / 2 / 0,9	1 / 1 / 1 / 1
271-360 min	0,51 / 0,29 / 0,12	Favourable outcome (mRS0-2) calculated based on probability		2 / 1,5 / 0,5	1 / 1 / 1 / 1
361-480 min		after Isch & Cons & Fav (0,48) + ARR			
Fav / Unfav / Death					
After Heam Fav / Unfav / Death		Anderson et al. 2013[7]			
	0,44 / 0,44 / 0,12	Control Group	Dirichlet	13 / 12 / 3	1 / 1 / 1 / 1
Utilities					
Utility in the favourable state (mRS0-2)	0,74	Dorman et al.1997[8]	Beta	103	36
Utility in the unfavourable state (mRS3-5)	0,38	Dorman et al.1997[8]	Beta	248	404
Utility in the death state	0				
Utility in the recurrent state	0,34	Morris et al.2011[9]	Beta	263	511,8
Markov transitions					
Mortality after stroke 70-74 years	0,05		Beta	294	812
Mortality after stroke 75-79 years	0,08	Based on Belgian mortality statistics	Beta	288	742
Mortality after stroke 80-84 years	0,14	multiplied with 2,5 (multiplier for age specific mortality among	Beta	277	630
Mortality after stroke 85-89 years	0,26	stroke patients)[10]	Beta	261	49
Mortality after stroke 90+ years	0,45		Beta	222	277

ARR= Absolute Risk Reduction, NNT = number needed to treat, ARD = Absolute Risk Difference, rpbl = rapid blood pressure lowering, Fav = favourable outcome (mRS 0-2), Unfav = unfavourable outcome (mRS 3-5), TIA = transient ischemic attack, Isch = ischemic stroke, Haem = haemorrhagic stroke, cons = conservative treatment, OTT = onset to treatment, IVT= intravenous administration of tissue Plasminogen Activator, EVT = endovascular treatment, min = minutes

1. Sheppard JP, Lindenmeyer A, Mellor RM, Greenfield S, Mant J, Quinn T, et al. Prevalence and predictors of hospital prealerting in acute stroke: a mixed methods study. Emerg Med J. 2016 Jul;33(7):482-8. PMID: 26949969. doi: 10.1136/emermed-2014-204392.
2. Vanacker P, Lambrou D, Eskandari A, Mosimann PJ, Maghraoui A, Michel P. Eligibility and Predictors for Acute Revascularization Procedures in a Stroke Center. Stroke; a journal of cerebral circulation. 2016 Jul;47(7):1844-9. PMID: 27301945. doi: 10.1161/strokeaha.115.012577.
3. Lees KR, Bluhmki E, von Kummer R, Brott TG, Toni D, Grotta JC, et al. Time to treatment with intravenous alteplase and outcome in stroke: an updated pooled analysis of ECASS, ATLANTIS, NINDS, and EPITHET trials. Lancet. 2010 May 15;375(9727):1695-703. PMID: 20472172. doi: 10.1016/s0140-6736(10)60491-6.

4. Campbell BC, Hill MD, Rubiera M, Menon BK, Demchuk A, Donnan GA, et al. Safety and Efficacy of Solitaire Stent Thrombectomy: Individual Patient Data Meta-Analysis of Randomized Trials. Stroke; a journal of cerebral circulation. 2016 Mar;47(3):798-806. PMID: 26888532. doi: 10.1161/strokeaha.115.012360.

5. Wardlaw JM, Murray V, Berge E, del Zoppo G, Sandercock P, Lindley RL, et al. Recombinant tissue plasminogen activator for acute ischaemic stroke: an updated systematic review and meta-analysis. Lancet. 2012 Jun 23;379(9834):2364-72. PMID: 22632907. doi: 10.1016/s0140-6736(12)60738-7.

6. Fransen PS, Berkhemer OA, Lingsma HF, Beumer D, van den Berg LA, Yoo AJ, et al. Time to Reperfusion and Treatment Effect for Acute Ischemic Stroke: A Randomized Clinical Trial. JAMA Neurol. 2016 Feb 1;73(2):190-6. PMID: 26716735. doi: 10.1001/jamaneurol.2015.3886.

7. Anderson CS, Heeley E, Huang Y, Wang J, Stapf C, Delcourt C, et al. Rapid blood-pressure lowering in patients with acute intracerebral hemorrhage. N Engl J Med. 2013 Jun 20;368(25):2355-65. PMID: 23713578. doi: 10.1056/NEJMoa1214609.

8. Dorman PJ, Waddell F, Slattery J, Dennis M, Sandercock P. Is the EuroQol a valid measure of health-related quality of life after stroke? Stroke; a journal of cerebral circulation. 1997 Oct;28(10):1876-82. PMID: 9341688.

9. Morris S, Hunter R, Davie C, Thompson A, Walker H, Thomson N, et al. Cost-effectiveness analysis of the london stroke service. University College London Report London, UK: University College London; 2011:1-62

10. Sandercock P, Berge E, Dennis M, Forbes J, Hand P, Kwan J, et al. Cost-effectiveness of thrombolysis with recombinant tissue plasminogen activator for acute ischemic stroke assessed by a model based on UK NHS costs. Stroke; a journal of cerebral circulation. 2004 Jun;35(6):1490-7. PMID: 15105519. doi: 10.1161/01.str.0000126871.98801.6e.
