# Supplementary material for: Time Gain Needed for In-Ambulance Telemedicine: Cost-Utility Model
Source: JMIR Mhealth Uhealth. 2017 Nov 24;5(11):e175. doi: 10.2196/mhealth.8288 (PMC5722977; doi:10.2196/mhealth.8288)
Supplement: Multimedia Appendix 2 [file mhealth_v5i11e175_app2.pdf]

Multimedia Appendix 2: Details on the costs used in the model

No	Parameter	Base-case value	Calculation/ Assumption/ Source	Distribution	Alpha	Beta
1	After mimic					
	Favourable	$5 351 (€4 027)	Values taken from UZ Brussel Stroke	Gamma	169	24
	Unfavourable	$33 333 (€25 082)	Registry linked with individual hospital	Gamma	400	63
	Death	$12 517 (€9 419)	claims data	Gamma	29	319
2	After TIA					
	Favourable	$7 042 (€5 299)	Values taken from UZ Brussel Stroke	Gamma	324	16
	Unfavourable	$20 660 (€15 546)	Registry linked with individual hospital	Gamma	73	214
	Death	$8 265 (€6 219)	claims data	Gamma	24	255
3	After Isch & Cons					
	Favourable	$13 690 (€10 301)	Values taken from UZ Brussel Stroke	Gamma	318	32
	Unfavourable	$39 435 (€29 673)	Registry linked with individual hospital	Gamma	827	36
	Death	$19 409 (€14 604)	claims data	Gamma	132	110
4	After Isch & IVT					
	Favourable	$18 797 (€14 144)	Values taken from Stroke UZ Brussel	Gamma	86	164
	Unfavourable	$45 862 (€34 509)	Stroke Registry linked with individual	Gamma	301	115
	Death	$18 311 (€13 778)	hospital claims data	Gamma	49	283
5	After Isch & EVT					
	Favourable	$24 823 (€18 678)		Gamma	400	46
	Unfavourable	$53 197 (€40 028)	(4) + (6)	Gamma	400	100
	Death	$24 606 (€18 515)		Gamma	400	46
6	Mean costs of Materials used in the operative room for EVT					
	Favourable	$6 025 (€4 534)		NA	NA	NA
	Unfavourable	$7 334 (€5 519)	Bing 2013 [1]	NA	NA	NA
	Death	$6 295 (€4 737)		NA	NA	NA
7	Additional cost of IVT	$561 (€422)	Cost of 50 ml Alteplase in Belgium – assume every patient is administered 50 ml	NA	NA	NA
8	After Haem & Cons					
	Favourable	$29 545 (€22 231)	Values taken from UZ Brussel Stroke	Gamma	64	346
	Unfavourable	$48 003 (€36 120)	Registry l linked with individual hospital	Gamma	329	110
	Death	$10 927 (€8 222)	claims data	Gamma	39	208

9	Costs of one year cycle (Markov Model)					
	Favourable	$4 115 (€3 096)	Health care utilization based on CERISE database[2] and UZ Brussel Stroke Registry More information in Multimedia Appendix 3	Gamma	400	8
	Unfavourable	$19 188 (€14 438)		Gamma	400	36
10	Costs of Hospitalization recurrent stroke (Markov Model)	$8 789 (€6 613)	Values taken from UZ Brussel Stroke Registry linked with individual hospital claims data	Gamma	1913	4
11	After Isch & IVT & Inatel		Weighted average between the cost for IVT (4) and the cost for conservative care (3) for each new patient receiving IVT summed with the additional cost of IVT (7) for each new patient.			
	Favourable	$16 812 (€12 650)		Gamma	400	32
	Unfavourable	$43 558 (€32 775)		Gamma	400	82
	Death	$19 010 (€14 304)		Gamma	400	36
12	After Isch & EVT & Inatel		Weighted average between the cost for EVT (5) and the cost for conservative care (3) for each new patient receiving EVT summed with the additional cost of EVT (6) for each new patient.			
	Favourable	$21 984 (€16 542)		Gamma	400	32
	Unfavourable	$50 131 (€37 721)		Gamma	400	82
	Death	$24 967 (€18 786)		Gamma	400	36

SE = Stander Error, rpbl = rapid blood pressure lowering, Favourable = favourable outcome (mRS0-2), Unfavourable = unfavourable outcome (mRS3-5), TIA = transient ischemic attack, Isch = ischemic stroke, Haem = haemorrhagic stroke, cons = conservative treatment, OTT = onset to treatment, IVT= intravenous administration of tissue Plasminogen Activator, EVT = endovascular treatment, NA= Non Applicable, Inatel = In-ambulance telemedicine

1. Bing F, Jacquin G, Poppe A, Roy D, Raymond J, Weill A. The cost of materials for intra-arterial thrombectomy. Interv Neuroradiol. 2013 Mar;19(1):83-6. PMID: 23472729.
2. Putman K, De Wit L, Schupp W, Baert I, Brinkmann N, Dejaeger E, et al. Variations in follow-up services after inpatient stroke rehabilitation: a multicentre study. Journal of rehabilitation medicine. 2009 Jul;41(8):646-53. PMID: 19565159. doi: 10.2340/16501977-0385.
