# Supplementary material for: Time Gain Needed for In-Ambulance Telemedicine: Cost-Utility Model
Source: JMIR Mhealth Uhealth. 2017 Nov 24;5(11):e175. doi: 10.2196/mhealth.8288 (PMC5722977; doi:10.2196/mhealth.8288)
Supplement: Multimedia Appendix 3 [file mhealth_v5i11e175_app3.pdf]

Multimedia Appendix 3: Details on health care utilization costs based on CERISE database[1] and UZ Brussel Stroke Registry– 1 year post-stroke costs

	Unit cost (1)	Mean absolute Frequency mRS 0-2 (2)	Relative Frequency mRS0-2 (3)	Mean absolute Frequency mRS 3-5 (4)	Relative Frequency mRS 3-5 (5)	**Total Cost mRS 0-2**	**Total Cost mRS 3-5**
GP visitis	€ 24,48	5,76	0,84	6.8	0,91	(1)*(2)*(3) = € 236,38	(1)*(4)*(5) = € 304,15
Specialist visits	€ 53,76	2,68	0,71	2,5	0,91	€ 204,11	€ 241
Physiotherpay	€ 22,26	23,93	0,73	26,8	0,67	€ 778,86	€ 794,61
Speech therapy	€ 22,05	14,75	0,22	20,0	0,27	€ 139,89	€ 240,55
Rehabilitation center costs	€ 18 250 (one year cost)	1	0,016*	1	0,5*	€ 292	€ 9125
Home nursing costs	€ 12 045 (one year cost)	1	0,12*	1	0,31*	€ 1445,4	€ 3734
**Total Costs**						**€ 3 096**	**€ 14 438**

Unit costs taken from Belgian RIZIV codes (payer + provider cost), all other data taken from CERISE database, *taken from UZ Brussel Stroke Registry
RIZIV = Rijksinstituut voor ziekte-en invaliditeitsverzekering, GP = General Practitioner,
Rounding of numbers reported in this table leads to a slightly different calculation then reported in Total Costs in the table

1. Putman K, De Wit L, Schupp W, Baert I, Brinkmann N, Dejaeger E, et al. Variations in follow-up services after inpatient stroke rehabilitation: a multicentre study. Journal of rehabilitation medicine. 2009 Jul;41(8):646-53. PMID: 19565159. doi: 10.2340/16501977-0385.
