# Supplementary material for: Time Gain Needed for In-Ambulance Telemedicine: Cost-Utility Model
Source: JMIR Mhealth Uhealth. 2017 Nov 24;5(11):e175. doi: 10.2196/mhealth.8288 (PMC5722977; doi:10.2196/mhealth.8288)
Supplement: Multimedia Appendix 4 [file mhealth_v5i11e175_app4.pdf]

Multimedia Appendix 4: Adapted probabilities for in-ambulance telemedicine

Parameter	Base-Case Value	Calculation/ Assumption/ Source	Distribution	Alpha	Beta
After Suspected Stroke<br>Standard Care / Inatel	0,61 / 0,39	The proportion of inclusions in the PreSSUB-I trial[12] divided by the total amount of registered patients during the same period in the UZ Brussel Stroke Registry(= 16/41 = 0,39%).	Dirichlet	610/490	1/1
after Isch<br>IVT / EVT / Cons	0,19 / 0,07 / 0,73	Compared to standard care (supplemental table 1), an absolute increase in the amount of IVT + EVT patients of 16% multiplied with time-gain/30 is modeled based on the information from Lorenz 2016[13]:<br>*'Probability that patient had dropped out of time window since prehospital evaluation*<br>*30 min = 16,2%'*<br>For 12 minutes time-gain an increase of 12/30*16.2% = 6.5% is modeled.<br>EVT assumed equal to eligible proportion reported in Vanacker et al. 2016[2] multiplied with time-gain/30. After 30 minutes time gain no additional effect is modelled.	Dirichlet	169 / 70 / 425	1 / 1 / 1
After Isch & IVT<br>0-90 min / 91-180 min / 181-270 min	0,15 / 0,29 / 0,56	Positive linear time shift of 12 min assumed compared to standard care. To apply time shift we assume patients are distributed equally in a time interval. A shift of 30 minutes in a time interval of 90 minutes, means that 1/3th (30min/90min) of patients within that time interval shift.	Dirichlet	25 / 44 / 67 / 34	1 / 1 / 1 / 1
after Isch & EVT<br>0-180 min /181-270 min / 271-360 min /361-480 min	0,19 / 0,23 / 0,38 / 0,19	Idem as above	Dirichlet	16 / 18 / 24 / 11	1 / 1 / 1 / 1

ARR= Absolute Risk Reduction, NNT = number needed to treat, ARD = Absolute Risk Difference, rpbl = rapid blood pressure lowering, Fav = favourable outcome (mRS 0-2), Unfav = unfavourable outcome (mRS 3-5), TIA = transient ischemic attack, Isch = ischemic stroke, Haem = haemorrhagic stroke, cons = conservative treatment,
OTT = onset to treatment, IVT= intravenous administration of tissue Plasminogen Activator, EVT = endovascular treatment, min = minutes, NA = Non-applicable, Inatel = in-ambulance telemedicine
