# Supplementary material for: Time Gain Needed for In-Ambulance Telemedicine: Cost-Utility Model
Source: JMIR Mhealth Uhealth. 2017 Nov 24;5(11):e175. doi: 10.2196/mhealth.8288 (PMC5722977; doi:10.2196/mhealth.8288)
Supplement: Multimedia Appendix 5 [file mhealth_v5i11e175_app5.pdf]

Multimedia Appendix 5: Costs related to in-ambulance telemedicine

Parameter	Base-Case Value	Calculation/ Assumption/ Source	Distribution	Alpha	Beta
Cost per teleconsultation	$143 (€107,52)	RIZIV nomenclature 102675 multiplied by 2 – we assume a teleconsultation will cost double a normal consultation	Gamma	400	0,13
Cost of installation of one telemedicine device	$34 554 (€26 000)	Based on commercial offer from Zebra Academy©:<br>Hardware cost = € 36.000<br>Service cost (including connectivity & training) = € 14.000<br>Hardware cost linearly amortized on 3 years (=12.000/year)<br>Summed service cost and amortized hardware cost to get installation cost	Gamma	400	65
Number of patients that can be treated with one device in one year	150	Conservative assumption based on activation rates of the PreSSUB-I trial[1] (3 activations per week)	NA	NA	NA
Estimated total cost for in-ambulance telemedicine for 1000 patients in one year	$159 425 (€119 959)	Number of installed ambulances*Cost of installation of one telemedicine device + number of patients in cohort*cost per teleconsultation<br>7*€26 000 + 1000*€53,76	NA	NA	NA

NA= Not Applicable, RIZIV = Rijksinstituut voor ziekte-en invaliditeitsverzekering

1. Valenzuela Espinoza A, Van Hooff RJ, De Smedt A, Moens M, Yperzeele L, Nieboer K, et al. Development and Pilot Testing of 24/7 In-Ambulance Telemedicine for Acute Stroke: Prehospital Stroke Study at the Universitair Ziekenhuis Brussel-Project. Cerebrovascular diseases (Basel, Switzerland). 2016;42(1-2):15-22. PMID: 26950076. doi: 10.1159/000444175.
