# Supplementary material for: Time Gain Needed for In-Ambulance Telemedicine: Cost-Utility Model
Source: JMIR Mhealth Uhealth. 2017 Nov 24;5(11):e175. doi: 10.2196/mhealth.8288 (PMC5722977; doi:10.2196/mhealth.8288)
Supplement: Multimedia Appendix 6 [file mhealth_v5i11e175_app6.pdf]

Multimedia Appendix 6. Tornado input probabilities – incremental cost per patient

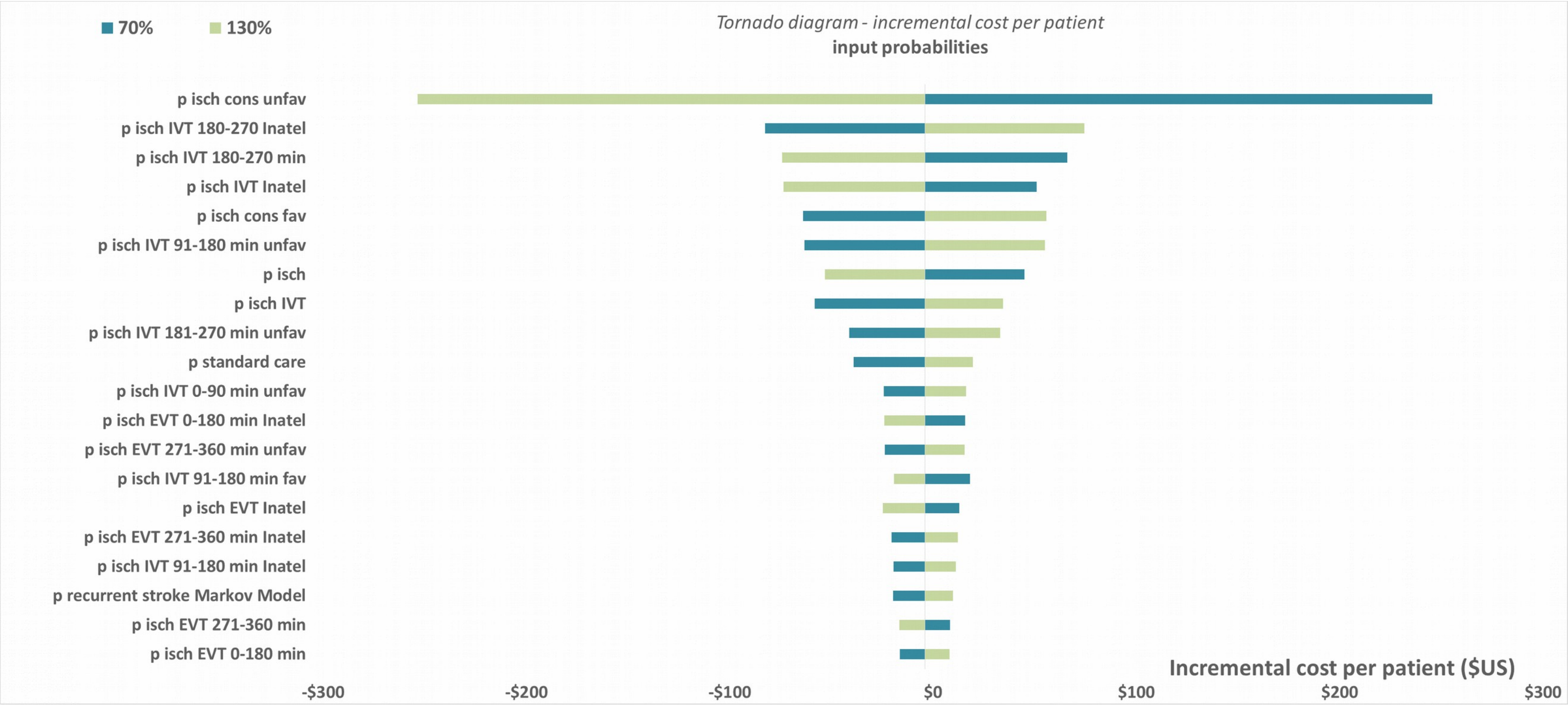

fav = favourable outcome (mRS 0-2), unfav = unfavourable outcome (mRS 3-5), isch = ischemic stroke, haem = haemorrhagic stroke, cons = conservative treatment, IVT= intravenous administration of tissue Plasminogen Activator, EVT = endovascular treatment, Inatel = In-ambulance Telemedicine, y = year, min= minutes, p = probability, standard = standard care (as a proportion of total patients vs. in-ambulance telemedicine)

Input parameters that lead to an effect of less than $20 dispersion per patient not shown here.

Model for 12 minutes time gain with in-ambulance telemedicine.
