# Supplementary material for: Time Gain Needed for In-Ambulance Telemedicine: Cost-Utility Model
Source: JMIR Mhealth Uhealth. 2017 Nov 24;5(11):e175. doi: 10.2196/mhealth.8288 (PMC5722977; doi:10.2196/mhealth.8288)
Supplement: Multimedia Appendix 8 [file mhealth_v5i11e175_app8.pdf]

Multimedia Appendix 8. Tornado input costs, utilities & other parameters – incremental cost per patient

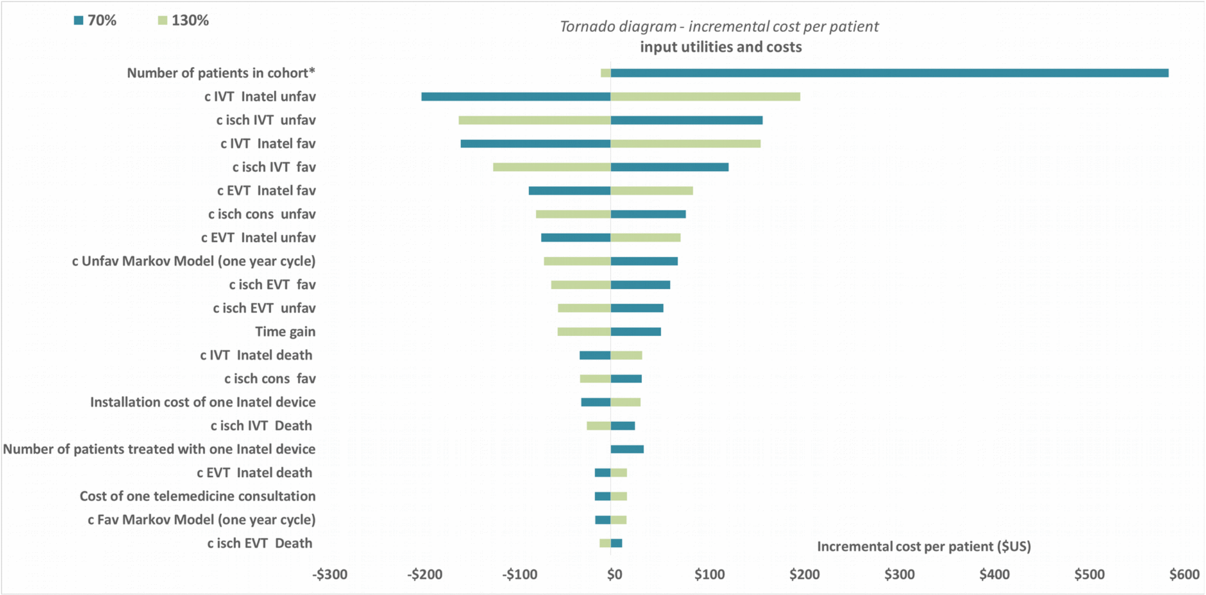

rpbl = rapid blood pressure lowering, Favourable = favourable outcome (mRS0-2), Unfavourable = unfavourable outcome, fav = favourable outcome (mRS 0-2), unfav = unfavourable outcome (mRS 3-5), isch = ischemic stroke, haem = haemorrhagic stroke, cons = conservative treatment, IVT= intravenous administration of tissue Plasminogen Activator, EVT = endovascular treatment, Inatel = In-ambulance Telemedicine, y = year, min= minutes, c = cost

*Number of patients in cohort varied to 50 (blue) & 10 000 (green)

Input parameters that lead to an effect of less than $20 dispersion per patient not shown here.

Model for 12 minutes time gain with in-ambulance telemedicine.
