# Supplementary material for: Time Gain Needed for In-Ambulance Telemedicine: Cost-Utility Model
Source: JMIR Mhealth Uhealth. 2017 Nov 24;5(11):e175. doi: 10.2196/mhealth.8288 (PMC5722977; doi:10.2196/mhealth.8288)
Supplement: Multimedia Appendix 9 [file mhealth_v5i11e175_app9.pdf]

Multimedia Appendix 9. Tornado input costs, utilities & other parameters – incremental QALY per patient

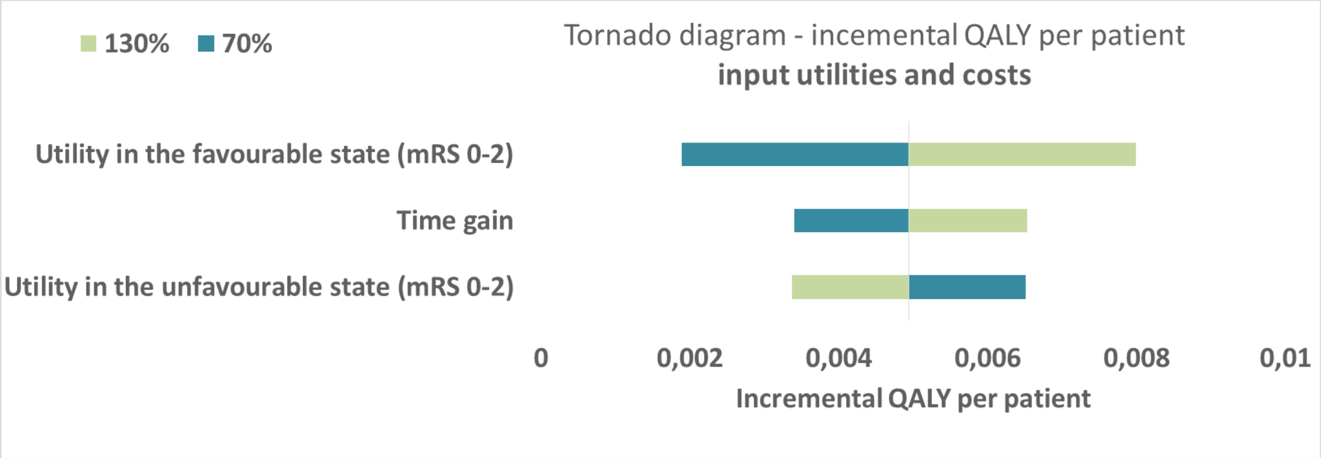

Input parameters that lead to an effect of less than 0,001 QALY dispersion per patient not shown her.

Model for 12 minutes time gain with in-ambulance telemedicine.
